# The Relation of Dependency and the Predictive Potential of Several Factors Possibly Involved in Determining Pulmonary Hypertension in Graves’ Disease

**DOI:** 10.12669/pjms.343.14500

**Published:** 2018

**Authors:** Camelia C. Scarneciu, Livia Sangeorzan, Mihaela Popescu, Vlad D. Scarneciu, Ioan Scarneciu

**Affiliations:** 1 Camelia C. Scarneciu, MD, PhD. Associate Professor, Fundamental Disciplines, Clinical Prevention Department, Transilvania University of Brasov, Romania; 2 Livia Sangeorzan, PhD. Associate Professor, Mathematics and Computer Science Department, Transilvania University of Brasov, Romania; 3 Mihaela Popescu, PhD. Lecturer, Department of Theoretical and Applied Linguistics, and Applied Linguistics, Transilvania University of Brasov, Romania; 4 Vlad D. Scarneciu, MD, PhD. Candidate. Interdisciplinary Doctoral School, Transilvania University of Brasov, Romania; 5 Ioan Scarneciu, MD, PhD. Associate Professor, Medical and Surgical Specialties Department, Transilvania University of Brasov, Romania

**Keywords:** Pulmonary hypertension, Graves’, disease, Non-linear model, Predictive potential

## Abstract

**Objectives:**

To establish a possible relation of dependency between pulmonary hypertension (PHT) and several factors, with the evaluation of their predictive potential, in Graves’ disease.

**Methods:**

For identifying the factors implied in producing PHT and for evaluating its reversibility, we made echocardiography exams, sessions of monitoring the blood pressure during 24 hours and biological test in a group of 42 patients with Graves’ disease (group H), comparing them with themselves in a euthyroid status (group E, n=25) and with a control group (group C, n=25). In order to analyse the relation of dependency between pulmonary hypertension (PHT) and the factors identified in the H group, we used both the simple linear regression method (polynomial of degree 1) and the non-linear regression method (polynomial of degree 2, 3) for establishing one model of functional dependency. We used the values of the coefficients of correlation *r* (degree of dependency) and of determination *R^2^* (the type of dependency). The statistical test (F-test, AIC criterion, test t) was applied by choosing the most appropriate model of determination, with a higher predictive potential.

**Results:**

We identified PHT at 47.6% of the patients with Graves’ disease. Once the euthyroidism status is obtained, PHT is normalized. While inducing PHT, we identified a strong relationship of dependency on several possible new factors such as: pre-treatment period, age, level of the thyroid stimulating hormone receptor antibody and values of systolic blood pressure, besides the already known ones (high level of thyroids hormones, cardiac output, pulmonary vascular resistance).

**Conclusions:**

The non-linear model best explains the relation of determination between pulmonary pressure and those factors having a better predictive potential (from 51% to 90%), compared with the linear model, the only exception being the age factor and the systolic blood pressure, where both models seems to be appropriate.

## INTRODUCTION

The last Guidelines for the Diagnosis and Treatment of Pulmonary Hypertension classifies pulmonary hypertension in hyperthyroidism in the 5^th^ category, with a multi-factorial mechanism, not well determined or even not clearly expressed.^1^ It recommends the determination of pulmonary hypertension by making the catheterization of the right heart and it specifies that the continuous wave Doppler only estimates the pulmonary hypertension.

The main hemodynamic determinants known to produce pulmonary hypertension in hyperthyroidism are increasing levels of thyroid hormones, pulmonary vascular resistance and high cardiac output.^2^-^4^ The excess of thyroid hormones leads to the increase of cardiac contractility, the increase of cardiac flow and of systolic pressure blood and decreases the systemic vascular resistance.^2^-^4^ The rise of CO is itself determined by the increase of HR and LVEF as an effect of the direct and indirect action of thyroid hormones and hyper-sympatheticotonia. In this context, the mechanism determining the increase of the pulmonary vascular resistance is not known, fact that can be clearly noticed in different studies.^3^-^8^ Several studies on Graves’disease show that the increase of pulmonary pressure depends on the increase of pulmonary vascular resistance a possible immune mechanism.^5^-^8^ The physiopathologic mechanism of producing pulmonary hypertension in hyperthyroidism is not clearly defined. There are studies that stipulate the normalizing of the values of pulmonary blood pressure once the clinic eutyroidism is obtained.^3^,^4^ Other studies reside in the evaluation of the relationship of correlation and of determination between different parameters and pulmonary hypertension by using the linear regression method.^3^,^4^,^6^,^8^

The purpose of this study was to identify the variables leading to a pulmonary pressure increase in Graves’ disease, and to determine the type of dependence relationship between them by using the linear and non-linear regression model, highlighting the predictive potential depending on the chosen model.

## METHODS

The study was approved by the Ethics Committee of Transilvania University of Brasov and conducted during 12 months, in 2015-2016. Newly-diagnosed Patients with Graves’ disease, who consecutively presented in the Endocrinology clinic and were under the age of fifty years, without history of cardiovascular, collagenous, pulmonary diseases were enrolled. Under these conditions, the cardiovascular changes should be strictly determined by hyperthyroidism.

Measurements were obtained at the time of admission (Hyperthyroid group [H group], n = 42) and after 12 weeks of follow up, when the euthyroid state is achieved (Euthyroid group [E group], n = 25). From the group of 42 patients with Graves’disease newly diagnosed, only 30 patients were present for the re-evaluation, and five out of them showed sub-clinic hyperthyroidism, consequently, there were only 25 patients in the group of euthyroidians. The results of these measurements were compared with those of a the 25 subjects, healthy, havin similar age and gender with the study group constituting the Control group (C group, n =25).

The diagnosis in Graves’ disease was established on those data supporting hyperthyroidism: free thyroxin level (FT_4_), with a value of higher than 23pmol/l, simultaneously with the low level of thyroid stimulating hormone (TSH) under 0.35µU/ml, in the presence of a higher level over 1.5UI/l of stimulating hormone receptor antibody (TRAb) and of a diffused goiter with increased blood output in the echographic examination of thyroid gland. Following the anamnesis, the pretreatment period from the beginning of the disease and its diagnosis was established.

Hormonal and immune determinations were made by utilizing an ARCHITECT analyzer Abbott Laboratories. Abbott Park, Illinois, USA. For TSH and FT_4_ a chemiluminescence immunoassay is used and for TRAb levels and enzyme-linked immunosorbent assay (ELISA) is used.

Echocardiography was performed by the same person with expertise in using Philips Sparq and Philips Sonos 7500 ultrasound machines (Philips, USA). The echographic measurements were made following standard procedures.^4^ Pulmonary artery systolic pressure (PAPs) was estimated by transtricuspidian flow velocity measurement and the variability of a diameter of inferior cava vein. Pulmonary artery mean pressure (PAPm) and pulmonary vascular resistance (PVR) was calculated by using Lindquist formula.^9^ For measuring the blood pressure and the ventricular frequency when the echocardiography was performed (useful for calculating the cardiac output (CO), an automatic device OMROM model HEM-FL31 (Japan was used. The ABPM/24-hour using Meditech ABPM-5 device (Hungary). In this study, we used only the mean systolic blood pressure/24-h (BP).

### Statistical analysis

In order to make a statistical analysis of data, we used the MedCalc Statistical Software version 17.9.7 (MedCalc Software bvba, Ostend, Belgium; http://www.medcalc.org; 2017).

The comparison of the mean values of the variables in the groups is made by applying the test t, which also allowed the calculation of statistical significance. Within the study, we considered as statistically significant a p<0.05 with 96% confidence interval (CI). We evaluated the relationship of dependency between these factors and the pulmonary pressure by determining the coefficients of correlation *r* (quantitative evaluation of the degree of dependency) and the coefficients of determination *R^2^* (evaluation dependency-type). The strength of association between 2 variables was calculated using the Pearson’s (*r*) (Normal distribution) or Spearman’s (*rs*) (non-uniform distribution) coefficient of correlation.

The coefficient of determination expresses the percentage of the PAPm’s variation, explained by each variable separately and express their strength of prediction (prediction’s potential). We calculate the coefficients of determination *R^2^* for the linear regression and non-linear regression models. The linear regression method based on an equation of 1^st^ degree with a straight line as a graphic expression. The linear regression model suggests the fact that the relationship of dependency is permanently either increasing or decreasing.

The non-linear regression models we chose as being the most convenient base on an equation of 2^nd^ degree, with a parabola as graphic expression and an equation of 3^rd^ degree, with a curve as a graphic expression. When choosing the non-linear regression model, we followed the graphic aspect of the curve and the possibility of its explaining from a physiopathological and clinical perspective. In order to validate each model of determination (regression model), we calculated the F-ration significance (p) of test F. Consequently, a p-value lower than 0.05 confirms the model’s validity, instead, a p-value higher than 0.05 makes it non-valid. To estimate the most appropriate model, the values of the coefficients of determination *R^2^* (*R^2^*-the highest^10^) are compared and then an analysis of residuals (sum of squares residual (SSE)-the lowest^10^) is made, when the values *R^2^* are visibly equal. We also applied an AIC-criterion (Akaike’s Information Criterion^10^) which takes into consideration the SSE and the numbers of parameters in the model; thus, we determine which of the two models is the most adequate.

## RESULTS

In Table-I, the main characteristics of the H group are presented to be compared with those of the C and E groups. As revealed in our patient group, Graves’ disease is approximately four times more frequent in women. We define as a pretreatment period, the period from the beginning of the symptomatology in hyperthyroidism to the diagnosis. This period is evaluated during several weeks and it is established through a rigorous anamnesis. Although, we assume that the patient might not make a precise appreciation by self-observation of this period. Pre-treatment period was approximately of 13.7 ± of 6.6 weeks (minimum two weeks and maximum 24 weeks). Both the low values of TSH and the high values of FT_4_ establish the diagnosis of hyperthyroidism. Their average values, within the three groups, are shown in Table-I. A TRAb level higher than 1,5 UI/l is a diagnosis for Graves’ disease. The median TRAb level H group was 62.4 ± 57 UI/l.(minimum value 4.3UI/l, maximum value 210UI/l) The hyperthyroid patients with Graves’ disease showed a significantly increased in LVEF, CO, Systolic BP/24h, PAPs, PAPm and PVR compared to patients in the E group and C group. There were no differences regarding average age, gender and the value of E/E’ ratio between the three groups. E and C groups are similar as referring to the average values of the entire number of factors we studied (Table-I).

**Table-I:** Main Characteristics in the Study Groups.

Parameter	(1) H group N = 42	(2) C group N = 25	p value (1/2)	(3) E group N = 25	p value (2/3)
Age (years)	34.8 ± 7.5	36.8 ± 8.9	0.328	34.8 ± 8.1	0.410
Women (%)	78.5	84	0.585	72	0.310
TSH(μU/ml)	0.03± 0.07	1.8± 0.7	< 0.0001	0.7± 0.2	< 0.0001
FT_4_ (pmol/l)	57.8 ± 24.1	15.3 ± 2.6	< 0.0001	15.2 ± 2.8	0.896
LVEF (%)	69.4 ± 4.7	66.7 ± 4.7	0.026	67.9 ± 4.9	0.381
CO (l/min)	6.6 ± 0.8	5.2 ± 0.5	< 0.0001	5.2 ± 0.5	1
E/E^’^	4.9 ± 1	4.7 ± 0.8	0.398	4.6 ± 0.7	0.640
PAPs (mmHg)	35.4 ± 7.3	24.2 ± 2.7	< 0.0001	24.1 ± 2.9	0.90
PAPm (mmHg)	23.6± 4.5	16.8± 1.7	< 0.0001	16.7± 1.7	0.836
PVR (WU)	2.1 ± 0.5	1.3 ± 0.4	< 0.0001	1.3 ± 0.4	1
BP (mmHg)	130 ± 14.2	106.5 ± 4.1	< 0.0001	106.2 ± 4.4	0.804

Note: H group - study group; C group – control group; E group – euthiroidian group; TSH-thyroid stimulating hormone; FT4-free thyroxin; TRAb-thyroid-stimulating hormone receptor antibody; LVEF-left ventricular ejection fraction; CO-cardiac output; E/E'-E wave peak velocity/E' wave maximal velocity; PAPs-systolic pulmonary arterial pressure; PAPm-median pulmonary arterial pressure; PVR-pulmonary vascular resistance; WU–Wood units; BP-mean systolic BP/24h.

The principle characteristics in the study group regarding the presence or absence in PHT are shown in Table-II. Thus, one can easily notice that, in the sub-group with PHT (PHT is defined as a PAPm≥25mmHg^1^) several parameters are at a high level and, consequently, they may determine the increase of pulmonary pressure. In addition, besides the already recognized parameters (CO, PVR)^2^-^4^,^6^,^8^ other parameters as TRAb level (as suggested in other studies^8^), age, pretreatment period, and the level of BP are identified.^4^ The result was unexpected. There was no difference between FT_4_ level within the two groups.

**Table-II:** Main Characteristics in the Study Group Regarding the Presence or Absence of PHT.

Parameter	(1) PHT Present (n=20)	(2) PHT Absent (n=22)	p
Age(years)	30.9±7.9	38.4±5.1	0.0007
Pretreatment period(weeks)	15.8±3.3	8.6±6.3	<0.0001
TSH(ìU/ml)	0.02±0.06	0.03±0.08	0.65
FT_4_(pmol/l)	56.7±22.4	58.7±26.1	0.79
TRAb(UI/l)	96.3±50.2	31.3±25.2	<0.0001
CO(l/min)	7.1±0.5	6.2±0.7	<0.0001
LVEF	72.9±2.5	66.2±3.8	<0.0001
E/E’	4.6±0.9	5.2±1	0.048
PAPs(mmHg)	41.9±4.5	29.5±3.0	<0.0001
PAPm(mmHg)	27.6±2.8	20.0±1.8	<0.0001
PVR(WU)	2.5±0.3	1.7±0.4	<0.0001
BP(mmHg)	140.4±10.8	120.5±9.6	<0.0001

Note: PHT Present-sub-group with pulmonary hypertension;

PHT Absent-sub-group without pulmonary hypertension; TSH-thyroid stimulating hormone;

FT_4_-free thyroxin; TRAb-thyroid-stimulating hormone receptor antibody; CO-cardiac output;

LVEF-left ventricular ejection fraction; E/E’-E wave peak velocity/E’ wave maximal velocity;

PAPs-systolic pulmonary arterial pressure; PAPm-median pulmonary arterial pressure;

PVR-pulmonary vascular resistance; WU–Wood units; BP-mean systolic BP/24h

The relation of association between PAPm and the studied variables, in conformity with the coefficient of correlation and of the corresponding level of statistical significance are shown in Table-III. The relations of association between PAPm with TRAb, PVRm, BP, CO and age, are very high and high, but mostly reasonable with the pretreatment period and non-existent with FT_4_ (fact clinically contradicted). The correlation of PAPm with age has a negative value.

**Table-III:** Association between mean pulmonary arterial pressure and studied parameters.

Parameters in correlation with PAPm	Pearson’s coefficient of correlation *r*	*p*-value	Spearman’s coefficient of correlation *rs*	*p*-value	Interpreting of correlation
FT4(pmol/l)			-0.24	0.125	Low correlation
TRAb(UI/l)			0.83	<0.0001	Very high correlation
Pretreatment period(weeks)			0.57	=0.0001	Reasonable correlation
Age(years)	-0.71	<0.0001			High correlation
PVR(WU)	0.92	<0.0001			Very high correlation
CO(l/min)	0.62	<0.0001			High correlation
BP(mmHg)	0.90	<0.0001			Very high correlation

Note: PAPm-median pulmonary arterial pressure; FT_4_-free thyroxin;

TRAb-thyroid-stimulating hormone receptor antibody; PVR-pulmonary vascular resistance;

WU–Wood units; CO-cardiac output; BP-mean systolic BP/24h.

The two proposals of regression models comparatively (exception being FT_4_, where three models are shown), linear (equation of 1^st^ degree) and parabola-type non-linear (equation of 2^nd^ degree), in order to estimate the relation of determination of PAPm by means of hemodynamic and non-hemodynamic parameters are shown in Table-IV. In order to determine the validity of each model, we used the test F^10^ and its level of significance for a p<0.05.

**Table-IV:** Comparison between linear and non-linear models of determining between predictor variables and PAPm (outcome variable).

Predictor variables	Regression Type	R^2^ [1]	SSE [2]	Significance F p value [3]	Model Validation [criterion]	Sign ΔAIC [4]	Preferred model of regression [criterion]	Predictive potential
FT_4_(pmol/l)	Linear	0.0001	548.6	0.96	Reject model [3]	(-)		0.01%
	Non-linear (2^nd^degree)	0.04	526.4	0.64	Reject model [3]			4%
	Non-linear (3^nd^degree)	0.51	270.8	0.001	Accept model [3]		Non-linear (3 degree) [1,2,4]	51%
TRAb(UI/l)	Linear	0.81	103.3	<0.0001	Accept model [3]	(-)		81%
	Non-linear (2^nd^degree)	0.84	87.3	<0.001	Accept model [3]		Non-linear [1,2,4]	84%
Pretreatment period(weeks)	Linear	0.12	485.6	0.09	Reject model [3]	(-)		12%
	Non-linear (2^nd^degree)	0.76	133.9	<0.0001	Accept model [3]		Non-linear [1,2,4]	76%
Age(years)	Linear	0.60	217.6	<0.0001	Accept model [3]	(+)	Linear [4]	60%
	Non-linear (2^nd^degree)	0.63	202.3	<0.0001	Accept model [3]		Non-linear [1,2]	63%
PVR(WU)	Linear	0.89	60.9	<0.0001	Accept model [3]	(-)		89%
	Non-linear (2^nd^degree)	0.90	54.6	<0.0001	Accept model [3]		Non-linear [1,2,4]	90%
BP(mmHg)	Linear	0.84	88.3	<0.0001	Accept model [3]	(+)	Linear [4]	84%
	Non-linear (2^nd^degree)	0.85	84.0	<0.0001	Accept model [3]		Non-linear [1,2]	85%
CO(l/min)	Linear	0.47	291.4	0.0002	Accept model [3]	(-)		47%
	Non-linear (2^nd^degree)	0.67	179.9	<0.0001	Accept model [3]		Non-linear [1,2,4]	67%

Note: FT_4_-free thyroxin; CO-cardiac output; BP-mean systolic BP/24h; PVR-pulmonary vascular resistance; R^2^-coefficient of determination;

[1]-criterion 1; SSE-sum of squares residual; [2]-criterion 2; Significance F=p value of significance of F-ratio;

[3]-criterion 3; Sign ΔAIC=Sign of the difference between AIC(Akaike’s Information Criterion) of the polynomial model and AIC of the linear model, if ΔAIC>0(+), the linear model is preferred, if ΔAIC <0(-) the nonlinear model is preferred;

[4]-criterion 4; Significance F (p<0.05)-significance level for F-ratio being a p<0.05.

In order to estimate the best model, we relied on several criteria, such as: the value of coefficient *R*^2^, the value of SSE^10^ and the test AIC^10^ with calculating ΔAIC. A higher value of *R^2^* and a lower value of SSE signify a better model. A ΔAIC>0 means that the simple model is better, ΔAIC<0 means that the complicated model is to prefer. Table-IV demonstrates that both regression models are accepted but the non-linear model is preferred, fulfilling all criteria regarding the determination between PAPm and TRAb, PVR and CO. As for the regression models between PAPm and Age, and BP, both models are accepted (*R^2^* and SSE support the non-linear model superiority; ΔAIC supports the linear model superiority). In case of regression between pretreatment period and PAPm only the non-linear model is accepted. In case of regression between FT_4_ and PAPm both regression models are invalidated. In this case, we applied a new non-linear regression model based on an equation of 3^rd^ degree and which is validated and seems to be an accepted model.

Thus, the predictive potential of FT_4_ increases, if we use the non-linear model, based on an equation of 3^rd^ degree. FT_4_ can explain 51% from the variation of PAPm in Graves’ disease. The predictive potential of the other predictor-variables is very important and higher for the non-linear model based on an equation of 2^nd^ degree (between 67% and 90%). This fact means that the predictor variables can explain between 67% and 90% from the variation PAPm in Graves’ disease. The predictive potential of variable predictors for PAPm is lower in the linear model, between 0.01% and 89%.

## DISSCUSION

We identified the presence of PHT in 47.6% of the patients with Graves’ disease in the study group, a percentage similar with the other studies.^3^-^6^,^8^ If most of the studies recognize the prevalence of pulmonary blood hypertension in hyperthyroidism, some of them consider that the mechanism of producing is due to the increase of the predominant cardiac-input^2^,^3^, instead, others notice the presence of a pathogenic relation^5^ between auto-immunity and HTP, by inducing apoptosis and pulmonary vascular remodeling^7^. Similar to our study, Sugiura^8^ highlights the fact that TRAb level is high when associated with the increase of pulmonary pressure, antibodies having an important role in endothelial influence and when the pulmonary vascular resistance increases in Graves’ disease.

PAPm≥25 mmHg under the conditions of LVEF>50% and E/E’ ratio<11 suggests that PHT is produced by means of a precapilary mechanism^1^. Once the status euthyroidism is obtained, the pulmonary values are normalized. (Table-I). From Table-II, we identified a range of factors possibly involved in producing PHT: some, already known, as the increased level of thyroid hormones, PVR and CO^2^-^4^,^6^,^8^; others suggested by studies, level of TRAb (as a marker of autoimmunity of Graves’ disease^8^) and a new the pretreatment period, age and the level of BP.^4^ By calculating the coefficients of correlation (Pearson and Spearman), we estimated the force of association between these variables and PAPm. We noticed the existance of a high and very high correlation for the relation of association of PAPm with TRAb, age, PVR, CO, BP and a reasonable one with pre-treatment period.

Surprisingly, we haven’t identified a correlation of PAPm with the thyroid hormones’ level, fact contradicting the clinical judgment. One can also notice that in other studies,^3^,^4^,^6^,^8^ similar to ours, the coefficients of correlation obtained in the linear regression between the thyroid hormones’ level and the pulmonary pressure in hyperthyroidism are lower and non-significant. The single explanation given for the fact that the increased level of the thyroid hormones is a principal and determining factor of pulmonary hypertension in hyperthyroidism was that the pulmonary hypertension appears in hyperthyroidism and disappears when euthyroidism is established.

After having identified a significant association between PAPm and the variables (factors) PVR, CO, BP, TRAb, pretreatment period and age, we determined the existence of a functional relation of determination by using the regression method. By calculating the coefficient of determination, we established the existence of a functional relation between all variables and PAPm. The non-linear regression model, an equation of 2^nd^ degree with a parabola as a graphic expression, is the accepted and most appropriate model for the relation of determination among TRAb, Pretreatment period, age, PVR, CO, BP and PAPm. In fact, the coefficient of determination expresses the percentage of the variation of PAPm, explained by the variation of each variable, taken separately. Within this model, in Graves’ disease, the prediction potential of each variable for the PAPm’s variation is very high, varying between 67% for CO and 90% for PVR. The linear model, though largely used in medical studies, proved not to be the most appropriate when expressing the functional relation of determination of PAPm in Graves’ disease. Nevertheless, the coefficients of determination calculated through the linear regression (equation of 1^st^ degree) are lower and has a reduced predictive value (between 12% for pretreatment period and 89% for PVR) than those calculate and PAPm becomes more complex and statistically significant only within a non-linear model expressed by an equation of 3^rd^ degree. The prediction value of FT_4_ for PAPm is of 0.01% for the linear model and 51% for the non-linear model (equation of 3^rd^ degree). In case of biological systems^10^, a functional relation expressed by an equation of 2^nd^ degree, having a parabola as a graphic expression better shows a modeling determined by the intervention of several mechanisms of adaptation, regulation and counter regulation than a relation expressed by an equation of 1^st^ degree.

### Limitations of the study

When related with the pulmonary pressure, expressed echographically and not by cardiac catheterism, as it is stipulated in the Guidelines for the diagnosis and treatment of pulmonary hypertension,^1^ and the study group is small.
